# Pin Tract Infections in Pediatric Open Long Bone Fractures: Common but Clinically Manageable

**DOI:** 10.3390/jcm15020525

**Published:** 2026-01-08

**Authors:** Britta Chocholka, Lara Marie Bogensperger, Vanessa Groß, Antonia Schwarz, Nicole Sophie Brunner, Manuela Jaindl, Stephan Payr

**Affiliations:** 1 University Clinic of Orthopedics and Trauma Surgery, Department of Trauma Surgery, Medical University of Vienna, Waehringer Guertel 18-20, 1090 Vienna, Austria; n11807478@students.meduniwien.ac.at (L.M.B.); n11916964@students.meduniwien.ac.at (V.G.); n12220006@students.meduniwien.ac.at (A.S.); nicole.brunner@meduniwien.ac.at (N.S.B.); manuela.jaindl@meduniwien.ac.at (M.J.); stephan.payr@meduniwien.ac.at (S.P.); 2 Section of Pediatric Trauma Surgery, Department of Trauma Surgery, University Clinic of Orthopedics and Trauma Surgery, Medical University of Vienna, 1090 Vienna, Austria

**Keywords:** pin tract infection, external fixation, pediatric trauma, open fractures, complications

## Abstract

**Background:** Pin tract infections (PTIs) are a frequent complication of external fixation, yet pediatric trauma-specific data—particularly for open long bone fractures—remain limited and heterogeneous. This study evaluated the frequency, severity, timing, management, and outcomes of PTIs in children and adolescents treated with external fixation for open long bone fractures. **Methods:** This retrospective single-center study included patients younger than 18 years with open long bone fractures treated with external fixation between 2002 and 2023. PTIs were graded using the Checketts–Otterburn classification (grades I–VI). Management included antibiotic regimen and surgical interventions. Outcome was reported by time to bony consolidation. **Results:** In 40 patients, 16 patients exhibited PTIs (mild: eight; moderate: five; severe: three. A higher grade of Gustilo–Anderson (*p* = 0.47) and evident macroscopic contamination (*p* = 0.73) did not appear to influence the occurrence of PTIs by similar duration of initial antibiotic regimen (*p* = 0.3). The median time to PTI onset was 49 days (IQR 22–80), with the majority occurring after completion of initial systemic antibiotic therapy. The management of PTIs was predominantly conservative: all eight mild cases resolved with intensified local pin tract care, while all eight moderate and severe cases were treated with systemic antibiotics and five required pin exchange or premature fixator removal. Overall bony consolidation was achieved in all patients, and reoperations were related to trauma severity rather than PTIs except in one patient. No cases of osteomyelitis were observed. **Conclusion:** Pin tract infections are frequently identified in pediatric open long bone fractures treated with external fixation. Using strict diagnostic criteria, any documented inflammatory change or local secretion at the pin–skin interface is considered indicative of PTI. However, the majority of these infections were classified as superficial and manageable with conservative measures, and all affected fractures healed radiologically.

## 1. Introduction

External fixation remains an essential technique for the stabilization of open long bone fractures in children and adolescents [1]. However, pediatric patients present unique challenges, including thinner soft tissue envelopes, higher contamination rates, and prolonged fixation duration, all of which increase the risk of pin tract infection (PTI) at the pin–skin interface [2,3]. It is an established fact that children demonstrate a remarkable biological capacity for fracture healing [4,5] and soft tissue regeneration [6,7]. The rich periosteal blood supply, high cellular activity, and pronounced remodeling potential of the growing skeleton might result in a relative resilience against progression from superficial infection to deep musculoskeletal involvement [8,9]. In this context, the classification proposed by Checketts and Otterburn [10] and later adopted by Dahl et al. [11], PTI represents a spectrum of inflammatory changes at the pin–skin interface, ranging from minimal erythema and serous discharge to deep infection involving soft tissue or bone, with even minor local signs considered indicative of infection [11,12]. Reported PTI rates vary widely in the literature, ranging from 9% to nearly 100%, largely depending on patient population, indication, and diagnostic criteria [3,13,14]. Open long bone fractures—particularly of the tibia—are especially prone to PTIs when managed with external fixation due to initial wound contamination, compromised soft tissue coverage, and the frequent need for staged procedures [15,16]. Adult trauma cohorts with open tibial fractures report PTI rates of approximately 20% to nearly 60%, substantially exceeding those observed in closed injuries [15,17]. Despite the clinical relevance of this complication, data focusing specifically on pediatric patients with open long bone fractures are limited. Most available studies primarily address elective limb lengthening or deformity correction [17], or combine adult and pediatric populations as well as open and closed fracture types [18], limiting their applicability to pediatric trauma care [19,20,21]. Therefore, this 20-year single-center retrospective study aims to determine the frequency, severity, risk factors, and outcome of pin tract infections in children and adolescents treated with external fixation for open long bone fractures.

## 2. Materials and Methods

This retrospective study was approved by the Ethics Committee of the Medical University of Vienna (EC-Code: 2075/2023) on 26 February 2025 and conducted in accordance with the Declaration of Helsinki and its latest amendments. All patients younger than 18 years who sustained an open long bone fracture between January 2002 and December 2023 and received treatment at the Level I Trauma Centre of the Department of Trauma Surgery at the Medical University of Vienna were screened for eligibility. Cases were identified using internal hospital databases and electronic medical records. Patients were included if they had at least one documented open long bone fracture of the upper or lower extremity that was initially stabilized with an external fixator. The exclusion criteria were missing documentation, pathological fracture etiologies, or the absence of external fixation as a treatment modality. Two patients were lost for follow-up and therefore excluded. For all eligible patients, demographic information (age at injury and sex), fracture characteristics (affected bone and classification according to Gustilo–Anderson), and the extent of soft tissue damage were extracted. Treatment-related parameters included timing and the type of external fixation, the number and mechanism (power drill or by hand) of applied pins, and the duration of external fixation, as well as the number and type of revision surgeries. Antibiotic administration, type of regimen, and duration of systemic prophylaxis in days were also recorded. Pin tract infections were graded according to the original Checketts–Otterburn classification (grades I–VI), as described by Checketts and Otterburn [10]. This system defines infection severity based on clinical findings at the pin–skin interface. For analytical purposes and due to the limited sample sizes within individual grades, infections were grouped into mild (grades I–II: localized erythema or discharge resolving with local care), moderate (grades III–IV: persistent drainage or tenderness requiring systemic antibiotics), and severe (grades V–VI: deep infection, or osteolysis requiring surgical intervention, pin exchange, or early fixator removal) categories. The outcome variables included the occurrence of complications such as PTIs, secondary displacement, delayed union, non-union, or malunion, as well as the clinical status at the last documented follow-up (FUP).

### Statistical Analysis

Descriptive analysis was performed for the entire cohort. In order to provide an epidemiological overview, the parameters described above were included. Continuous variables were presented depending on data distribution as mean values with standard deviation (SD) and ranges or as median with interquartile range (IQR). Categorical variables were reported as absolute frequencies and percentages. Comparisons between patients with and without pin tract infections were performed using Welch’s *t*-test for normally distributed continuous variables and the Mann–Whitney U test for non-normally distributed variables. Normal distribution was assessed using the Shapiro–Wilk test. Categorical variables were compared using Fisher’s exact test. All tests were two-sided, and statistical significance was set at a level of *p* < 0.05. The Bonferroni–Holm correction was performed for multiple testing. All statistical analyses were conducted using Microsoft^®^ Excel for macOS (Version 16.42; Microsoft Corp., Redmond, WA, USA) and IBM^®^ SPSS^®^ Statistics (Version 27.0.0; IBM Corp., Armonk, NY, USA).

## 3. Results

The study cohort comprised 40 pediatric patients with open fractures of the long bones who were treated with external fixation, including 25 boys and 15 girls. The overall mean age was 11.8 ± years (range: 4–17 years), with boys averaging 12.1 ± 4.2 years and girls 11.2 ± 4.0 years.

### 3.1. Injury Mechanisms and Fracture Distribution

In total, 28 patients (male: 19; female: 9) were involved in traffic accidents and 6 fell from higher than 2 m (male: 2; female: 4). Other injury mechanisms involved agricultural or industrial machinery, heavy equipment-related incidents, a train-related accident, and a work-related injury, alongside single cases of a bicycle crash and a fall from a horse (n = 6; m: 4; female: 2).

Across the 40 documented open long bone fractures, the lower leg was the most frequently affected site, with 28 tibial or tibia–fibula injuries (male: 15, female: 13), followed by eight femoral fractures (male: 7, female: 1), three open humeral shaft fractures (male: 2, female: 1), and one isolated forearm (n = 1; male).

### 3.2. Gustilo–Anderson Classification

In total, 11 fractures were classified as Grade I (male: 4, female: 7) and 13 as Grade II (male: 9, female: 4). Grade IIIa/IIIb open fractures occurred in 14 patients (male: 10, female: 4). In addition, two fractures in the dataset represented Grade IIIc injuries (both male).

The wound sizes of all 40 open fractures were documented as the following: nine wounds measuring ≤ 1 cm, 17 wounds between 1 and 10 cm, ten wounds 10–30 cm, and four wounds > 30 cm. Wound contamination was reported in 12 patients most commonly due to road debris (n = 8), soil contamination (n = 1) and other miscellaneous sources (n = 3).

### 3.3. Initial Antibiotic Therapy and Management

All 40 children received systemic antibiotic prophylaxis within the first hour of admission. The most frequently administered regimen was a combination of amoxicillin-clavulanic acid and gentamicin (n = 24), followed by cefuroxime (n = 8) and ampicillin-sulbactam (n = 6). Two children received alternative regimens, one with ciprofloxacin and one with amoxicillin–clavulanic acid alone.

In detail, in the PTI group (n = 16), the most frequently administered regimen was a combination of amoxicillin–clavulanic acid and gentamicin (n = 7), followed by cefuroxime only (n = 4). One patient received ampicillin–sulbactam combined with amoxicillin–clavulanic acid, one patient received ampicillin–sulbactam combined with gentamicin, and one patient received ampicillin–sulbactam as monotherapy. One patient each received ciprofloxacin (n = 1) or amoxicillin–clavulanic acid monotherapy (n = 1).

In the non-PTI group (n = 24), amoxicillin–clavulanic acid combined with gentamicin was likewise the most common initial regimen (n = 17), followed by monotherapy with cefuroxime (n = 4) or ampicillin–sulbactam (n = 3).

The duration of systemic antibiotic therapy was documented in 37/40 patients, with a mean of 9.9 ± 7.4 days (median 7 days; range 3–30 days). The duration of initial antibiotic therapy was comparable between patients with and without pin tract infections, with a mean treatment duration of 11.7 ± 8.5 days in the PTI group and 8.7 ± 7.1 days in patients without PTI, showing no statistically significant difference between groups (*p* = 0.30). (Table 1).

The interval from hospital admission to initial surgical management was 1.4 ± 1.1 h on average (median 1.0 h; range 0.5–4 h). Postoperative intensive care was required in 11 children (male: 7, female: 4), whereas 29 patients were treated on standard wards (male: 18, female: 11).

### 3.4. External Fixation Details and Wound Management

External fixation consisted most frequently as unilateral fixators (n = 24), followed by hybrid constructs (n = 13) and circular frames (n = 3). In addition to external fixation, three fractures were treated with additional Kirschner wires, and there were two single cases of additional plating or screw fixation. Most constructs were stabilized with four pins (n = 25), while six-pin (n = 8) and five-pin configurations (n = 6) were less common, and a single construct was applied using two pins. Pin placement was predominantly performed using a power-driven technique (n = 35), with manual insertion documented in five cases. Most pins had a diameter of 4 mm (n = 24), followed by 5 mm pins (n = 14) and, less commonly, 3 mm pins (n = 2).

Primary surgical wound closure was achieved in 24 of 40 patients, while 16 children required alternative or staged soft tissue management. Among these, split-thickness skin grafting was performed in seven patients, and flap coverage in four. Artificial skin substitutes were applied in two children. Two patients required fasciotomy for evolving compartment syndrome, and one child underwent amputation due to the severity of soft tissue injury.

### 3.5. Pin Tract Infections

A total of 16 patients (40% of the cohort) developed PTIs (male: 12, female: 4). Most infections arose in fractures of the lower leg (11/16; 68.8%), while the femur was affected in three cases (3/16; 18.7%) and the upper arm and forearm in one case each (2/16; 12.5%).

When stratified by soft tissue injury severity according to the Gustilo–Anderson classification and contamination, pin tract infections occurred in 3 of 11 patients (27.3%) with Gustilo–Anderson type I fractures and in 13 of 29 patients (44.8%) with Gustilo–Anderson type II-III fractures. The difference between groups was not statistically significant (*p* = 0.47). Macroscopically, apparent wound contamination was present in four (25%) patients with grade II, III, and two IIIc injuries. The remaining 12 infections occurred in grade I injuries without macroscopical contamination. The occurrence of pin tract infections did not differ significantly between macroscopically contaminated and non-contaminated wounds (*p* = 0.73).

### 3.6. Classification of PTIs According to Checketts–Otterburn

According to the Checketts–Otterburn classification, eight of the sixteen pin tract infections were classified as mild (grades I–II), five as moderate (grades III–IV), and three (18.8%) as severe (grades V–VI) (Table 2).

Overall, microbiological samples were obtained from seven of these patients and five were identified to have pathogen growth. Stratified according to the Checketts–Otterburn classification, culture positivity increased with infection severity (one grad II, moderate III–IV: 2, and severe V–VI: 2). Samples were taken in all three severe cases and three of five moderate cases and one mild case. Identified pathogens included Staphylococcus aureus (n = 1), beta-hemolytic streptococci (n = 1), Candida albicans (n = 1), mixed bacterial flora including coagulase-negative staphylococci and Enterococcus species (n = 1), and Acinetobacter/Corynebacterium species (n = 1).

When pin tract infections were present, severe infections may be associated with higher injury severity. Gustilo–Anderson type III fractures represented two of the three severe PTIs, two of the five moderate, and two of the eight mild infections. The initial mean wound size ranged from 3.5 ± 3.2 cm in mild PTIs, to 10.0 ± 5.2 cm in moderate and to 20.0 ± 12.0 cm in severe PTIs.

The median time from injury to infection was 49 days (IQR 22–80 days). When stratified according to the Checketts–Otterburn classification, infections occurred with a median time to onset of 49 days (IQR 32–86 days) for mild infections (grades I–II), 75 days (IQR 40–103 days) for moderate infections (grades III–IV), and 9 days (IQR 7–14 days) for severe infections (grades V–VI). Regarding systemic antibiotic therapy, two patients (12.5%) developed a pin tract infection while still receiving antibiotics, whereas fourteen of sixteen patients (87.5%) presented after cessation of antibiotic treatment. Two markedly delayed infections were documented at day 150 and day 405 post injury and were therefore analyzed separately; both occurred in severely polytraumatized patients with prolonged overall treatment courses.

### 3.7. Documented Pin Loosening

Pin loosening was documented in three cases, all of which occurred in patients with one moderate and two severe infections. Clinically relevant partial or progressive pin loosening of one or more pins in this cohort was limited to infections of intermediate and severe manifestation. All mild cases explicitly documented stable pins.

### 3.8. Treatment of the PTIs Stratified by Severity

Mild pin tract infections were managed conservatively; no hardware intervention was necessary in those eight patients. Six were treated with intensified local pin care alone, while two received short courses of antibiotics, including one patient treated with systemic amoxicillin–clavulanic acid for 5 days and one patient receiving topical antibiotic treatment only.

In contrast, all moderate infections (5/5) were treated with systemic antibiotics including amoxicillin–clavulanic acid (n = 3), first-generation cephalosporins (n = 1), or clindamycin (n = 1), with treatment durations ranging from 5 to 14 days (median 7 days). Hardware management was required in three of five cases, including pin exchange (n = 2) or premature fixator removal (n = 1) due to local instability or persistent inflammation.

Among severe infections, two of three patients required escalated treatment with early external fixator removal in addition to systemic antibiotic therapy, including amoxicillin–clavulanic acid or combination regimens with treatment durations of up to 30 days. In the remaining patient, a Checketts–Otterburn grade VI infection occurred after hardware removal and the surgical curettage of the pin tract with sequestrum removal was necessary subsequently.

### 3.9. Radiological Bony Consolidation

Bony consolidation in the PTI group was achieved in a median time of 16 weeks (IQR 11–20; range 5–66) whereas it took 9 weeks (IQR 8–11; range 6–21) in the non-PTI group. Stratified by infection severity, the median consolidation time was 14 weeks (range 5–23) in mild infections (n = 8), 18 weeks (range 5–53) in moderate infections (n = 5), and 53 weeks (range 5–66) in severe infections (n = 3).

Regular radiological consolidation was observed 62.5% (10/16) of PTIs (mild: 6/8, moderate: 3/5 moderate, and severe: 1/3). Delayed union occurred in 2/8 mild and 2/5 moderate cases, whereas pseudarthrosis was exclusively observed in 2/3 severe infections which were also consolidated at last FUP.

In the non-PTI group, regular consolidation was observed in 19 of 24 patients (79.2%), while delayed union occurred in five cases (20.8%). No cases of pseudarthrosis were documented in the non-PTI cohort.

## 4. Discussion

Pin tract infections are among the most frequently reported complications associated with external fixation; however, their clinical relevance in pediatric trauma—particularly in the setting of open long bone fractures—has not been comprehensively studied. While numerous publications describe PTIs in elective pediatric limb lengthening or deformity correction, trauma-related evidence in children remains limited and is often extrapolated from adult cohorts or mixed fracture populations [3,14,15]. The present study addresses this gap by providing a detailed analysis of PTI frequency, severity, timing, management, and outcomes in a well-defined cohort of children and adolescents treated with external fixation for open long bone fractures over a 20-year period.

In this present cohort, PTIs were documented in 40% of patients. At first glance, this incidence may appear high; however, it must be interpreted in the context of the strict classification criteria applied. In accordance with the Checketts–Otterburn system, any documented inflammatory change at the pin–skin interface—including minimal erythema or local secretion—was classified as a PTI, even in the absence of microbiological confirmation [12]. Consequently, some low-grade PTIs, particularly those characterized by minimal clear discharge without systemic or progressive local signs, may not necessarily represent true bacterial infection. Nevertheless, this conservative and clinically oriented approach is given to ensure early detection, standardized grading, and comprehensive reporting of all pin tract abnormalities. Given the substantial heterogeneity in PTI definitions across the literature, the wide range of reported infection rates—from below 10% to nearly 100%—likely [17,22,23] reflects differences in diagnostic thresholds rather than true variability in infection burden.

Despite the relatively high overall PTI rate, clinically relevant infections in this cohort were predominantly mild or moderate, while severe infections were distinctly rare. Importantly, no cases of osteomyelitis or chronic deep bone infection were observed, even in children with high-energy trauma, severe soft tissue injury, or advanced PTI grades. This finding supports previous pediatric observations suggesting that PTIs in children most often remain superficial and respond well to conservative measures [18], and contrasts with adult trauma cohorts—particularly those involving open tibial fractures—where higher rates of deep infection and osteomyelitis have been reported [15]. These differences may reflect both biological advantages present in children, such as superior soft tissue vascularity and healing capacity, and differences in injury patterns and comorbidities between pediatric and adult populations. Furthermore, the discrepancy between the findings of the present study and from adult cohorts may, at least in part, be explained by fundamental biological differences in the growing skeleton. Experimental research on bone regeneration highlights the importance of the local biological microenvironment, angiogenesis, and osteogenic signaling in maintaining fracture healing even in the presence of local inflammatory stimuli. Although such studies are not directly related to pin tract infections, they provide important biological context and may help explain why superficial infections did not compromise bone consolidation in the pediatric cohort [24,25].

The timing of PTI onset in the cohort further supports the concept that many infections represent superficial or early inflammatory processes rather than established bacterial infections. Most PTIs occurred after completion of the initial systemic antibiotic regimen and during early fixator retention, whereas only two patients developed PTIs while still receiving antibiotics. This observation suggests that early postoperative pin tract changes do not necessarily reflect antibiotic failure, but may instead represent local tissue response or mechanical irritation at the pin–skin interface [2]. Markedly delayed PTIs were observed only in two severely polytraumatized patients with exceptionally prolonged fixation periods and complex clinical courses, indicating that atypical infection timing may be more closely related to overall injury severity and treatment duration than to pin tract behavior alone.

A subsequent analysis of potential risk factors did not reveal a significant association between the occurrence of PTI and fracture severity or macroscopic wound contamination. A notable observation is that the duration of the initial antibiotic treatment does not seem to exert an influence on the incidence of PTI. Furthermore, the fracture location and pin insertion technique did not reveal any significant influence. Although previous studies have suggested that hand-driven pin insertion may reduce thermal necrosis and lower infection rates [3,26,27], the present data did not demonstrate the protective effect of this technique. However, given the small number of hand-driven pins and the uneven distribution across groups, these findings should be interpreted cautiously and considered to be hypothesis-generating rather than conclusive. Overall, the results highlight the complexity of PTI pathogenesis and underscore the need for larger, prospective pediatric studies to better delineate modifiable risk factors.

From a clinical perspective, PTI management in this cohort was predominantly conservative. Most mild infections resolved with intensified local pin tract care alone, while moderate infections were successfully treated with short courses of systemic antibiotics. Only a minority of patients required pin exchange or premature fixator removal, and no cases necessitated extensive surgical debridement or complex reconstructive procedures. Importantly, fixator stability was generally preserved, and intravenous antibiotic therapy was sufficient when required. These findings reinforce the concept that pediatric PTIs rarely necessitate aggressive surgical management and that early recognition combined with structured local care remains the cornerstone of treatment [3,17,23,28].

Pin loosening—often considered a hallmark of more advanced PTI—was documented in only three patients, all of whom had moderate or severe infections. Even in these cases, pin loosening did not progress to deep infection or osteomyelitis. This observation further supports the notion that pediatric patients possess a favorable biological environment that may mitigate progression from superficial pin tract irritation to more severe complications, even when local mechanical stability is partially compromised [3].

The overall outcomes in children who developed PTIs were favorable. Radiographic abnormalities such as delayed union or pseudarthrosis occurred in a minority of cases and were not clearly attributable to PTIs.

A limitation of this study is its retrospective design, which introduces issues related to missing data, their interpretation, and influence on certain parameters, such as microbiological findings and pin insertion techniques. Furthermore, the sample size appears to be relatively small, which is due to the monocentric design. The limited sample size of this study may constrain the investigation of further potential risk factors. The antibiotic regimens, whether preventive or therapeutic, were thus presented in a descriptive manner. Furthermore, statistical analysis did not suggest an association between PTI and fracture localization, wound contamination, or pin insertion technique. Despite the implementation of appropriate statistical methodologies, a degree of caution should be exercised when interpreting the findings, as they may be attributable to a limited capacity to discern potential statistical differences in this retrospective cohort study. However, considering the strict focus on pediatric patients and the highly unique nature of the topic, the sample size presented is still notably substantial compared to the current literature.

Despite these encouraging findings, the heterogeneity in pin care protocols, antibiotic regimens, and documentation observed over the long study period underscores the need for pediatric-specific standards. Standardized recommendations regarding pin insertion technique, pin tract care protocols, the duration of antibiotic prophylaxis, and early recognition of PTI severity could help reduce variability in clinical practice and improve consistency of care [14]. The structured education of caregivers and children regarding pin tract hygiene and monitoring may further enhance adherence and early detection of complications [18,19].

Finally, this study highlights the complexity of PTI pathogenesis and underscores the need for future prospective research focusing specifically on pediatric trauma populations. Most existing PTI literature is derived from adult cohorts or elective orthopedic procedures, which differ substantially from children with high-energy open fractures. Establishing pediatric-specific risk profiles, prevention strategies, and standardized treatment algorithms will be essential to further optimize the management of PTIs in this vulnerable population.

## 5. Conclusions

Pin tract infections are a frequent finding in children and adolescents treated with external fixation for open long bone fractures when strict, clinically oriented diagnostic criteria are applied. However, the vast majority of PTIs are low grade, respond well to conservative management, and do not progress to deep infection or osteomyelitis. The overall clinical and functional outcomes in affected children are favorable, and PTIs rarely necessitate aggressive surgical intervention. These findings emphasize that early recognition, structured pin tract care, and severity-adapted treatment are key to successful management. Future pediatric-specific guidelines and prospective studies are needed to further standardize prevention and treatment strategies for PTIs in pediatric trauma care.

## Figures and Tables

**Table 1:** Characteristics of the cohort.

Characteristics	Total n = 40	Non-PTIs n = 24	PTIs n = 16	*p*-Value
**Gustilo–Anderson**				*p* = 0.47
Grade I	11	8	3	
Grade II	13	6	7	
Grade III a/b	14	10	4	
Grade III c	2	0	2	
**Wound contamination**	12	8	4	*p* = 0.73
**Wound Size 3**				
≤1 cm	9	6	3	
1–10 cm	17	11	6	
10–30 cm	10	6	4	
>30 cm	4	2	2	
**Initial antibiotic treatment**				*p* = 0.3
Mean duration ± SD	9.9 ± 7.4	8.7 ± 7.1	11.7 ± 8.5	
**Fracture localization**				
Tibia/Fibula	28	17	11	
Femur	8	5	3	
Upper extremity	4	2	2	
**Management**				
Primary wound closure	24	14	10	
Staged management	16	10	6	

The duration of initial antibiotic therapy was documented in 37 of 40 patients. Wound contamination refers to macroscopically visible contamination documented at initial surgical exploration.

**Table 2:** Course of pin tract infections stratified by Checketts–Otterburn severity.

Therapy Modality	Mild (I–II) n = 8	Moderate (III–IV) n = 5	Severe (V–VI) n = 3	Total
Antibiotic therapy only	3	2	-	5
Local pin tract care only	5	-	-	5
Pin exchange	-	2	-	2
Fixator removal	-	1	2	3
Curettage/Sequestrectomy	-	-	1	1
Pin loosening	-	1	2	3
Delayed consolidation	2	2	2	6
Regular consolidation	6	3	1	10
Median time of bony consolidation in weeks	14	18	53	16

## Data Availability

The datasets generated and/or analyzed in the current study are not publicly available due to data privacy but are available from the corresponding author on reasonable request.

## Abbreviations

The following abbreviations are used in this manuscript:

FUP	Follow Up
IQR	Interquartile Range
PTI	Pin Tract Infection
SD	Standard Deviation
